# See and Strike: A Dual-Force Paradigm for Real-Time Lung Cancer Diagnosis and Non-Thermal Ablation

**DOI:** 10.3390/diagnostics16101553

**Published:** 2026-05-20

**Authors:** Jaskiran Khosa, Roy J. Cho

**Affiliations:** Division of Pulmonary, Allergy, Critical Care and Sleep Medicine, Section of Interventional Pulmonology, University of Minnesota, Minneapolis, MN 55455, USA; khosa011@umn.edu

**Keywords:** dynamic cell imaging (DCI), full-field optical coherence tomography (FF-OCT), pulsed electric field (PEF) ablation, non-thermal tumor ablation, bronchoscopic intervention, robotic-assisted bronchoscopy, lung cancer diagnostics, non-small cell lung cancer (NSCLC), real-time tissue viability assessment, intra-procedural pathology, precision oncology, minimally invasive tumor therapy, optical imaging, electroporation, bronchoscopic tumor ablation

## Abstract

Lung cancer remains the leading cause of cancer-related mortality worldwide despite advances in screening, navigational bronchoscopy, and systemic therapies. Diagnostic and therapeutic limitations persist, including uncertainty regarding intraprocedural tissue adequacy during biopsy sampling and constraints of existing ablative modalities for tumors located near critical thoracic structures. This review examines two emerging technologies: Full-Field Optical Coherence Tomography-based Dynamic Cell Imaging (DCI) and monopolar biphasic Pulsed Electric Field (PEF) ablation as complementary emerging technologies that may address these gaps. The Van Gogh™ Microscopy System (CellTivity Scientific, Inc.) utilizes DCI to enable real-time visualization of cellular metabolic activity without tissue destruction, providing functional information regarding tissue viability and microstructural morphology. The Aliya^®^ PEF ablation system (Galvanize Therapeutics, Inc.) delivers biphasic high-voltage electrical pulses that induce non-thermal tumor cell death while preserving extracellular matrix architecture, potentially allowing treatment near sensitive thoracic structures such as airways, vasculature, and pleura. Early preclinical studies and initial clinical experience suggest that DCI can facilitate rapid intraprocedural assessment of biopsy adequacy, while PEF ablation may provide reproducible focal tumor destruction with a favorable safety profile near critical structures. Although the current evidence base remains limited to early-phase studies and feasibility trials, the convergence of real-time biologic tissue assessment with structurally preserving ablation technologies introduces the possibility of integrating diagnostic confirmation and local therapy within a single procedural workflow. This review summarizes the mechanistic rationale, emerging evidence, and potential clinical applications of these technologies and proposes a conceptual “See and Strike” framework within these two emerging technologies. The methodological limitations, workflow considerations, and future research directions required to validate this approach are also discussed. Prospective multicenter trials and long-term oncologic outcomes will be necessary before widespread clinical adoption.

## 1. Introduction

Advances in bronchoscopic technology have expanded the ability to detect and localize pulmonary lesions with increasing precision; however, a critical gap remains between lesion identification and definitive, real-time treatment. In current practice, tissue diagnosis and therapeutic intervention are often temporally and procedurally separated, requiring multiple procedures, delayed decision-making, and potential loss to follow-up. This gap is particularly relevant in clinical scenarios such as early-stage lung cancer in non-surgical candidates, oligometastatic disease, and indeterminate pulmonary nodules, where timely diagnosis and localized therapy are essential for optimal outcomes [1,2,3,4].

A “See and Strike” strategy is the holy grail where technologies seek to integrate real-time histologic confirmation with immediate targeted therapy within a single procedural setting. This paradigm is especially valuable when (1) diagnostic uncertainty limits immediate treatment decisions, (2) patients are poor surgical candidates, or (3) lesion accessibility is challenging and repeat procedures carry increased risk. By enabling simultaneous visualization, characterization, and treatment, this approach has the potential to improve procedural efficiency, reduce healthcare utilization, and enhance patient-centered care [5,6,7].

Despite advances in navigation and localization technologies, current bronchoscopic workflows still rely heavily on delayed pathology results and staged interventions. Rapid on-site evaluation (ROSE) offers limited cytologic feedback and lacks the architectural and functional detail necessary for confident, real-time therapeutic decisions. Similarly, existing ablative modalities—such as thermal techniques (e.g., radiofrequency, microwave, and laser)—are constrained by collateral tissue injury and limited ability to preserve surrounding structures [8,9,10]. These limitations underscore the need for technologies that provide both high-resolution, real-time tissue characterization and precise, non-destructive therapeutic capabilities.

In this context, emerging technologies such as Dynamic Cell Imaging (DCI) and Pulsed Electric Field (PEF) ablation represent complementary innovations that together enable a one-stop shop approach. DCI, a form of dynamic full-field optical coherence tomography, provides label-free, real-time visualization of cellular architecture and metabolic activity while preserving tissue for downstream analysis. This allows for immediate assessment of tissue viability and malignant potential without the delays associated with conventional histopathology [11,12,13]. PEF ablation, in contrast, is a non-thermal modality that induces cell death through membrane disruption while preserving extracellular matrix and critical structures such as airways and vasculature. This property makes it particularly well-suited for use in delicate pulmonary environments where structural preservation is essential [14,15,16].

These two technologies were selected for this review because they are rapidly emerging within the field of thoracic oncology as potential gamechangers and uniquely address real-time diagnostic confidence and precision, tissue-sparing therapy. Together, they represent a shift from sequential to integrated procedural care. Importantly, they also occupy a distinct position within the broader spectrum of emerging technologies—bridging the gap between purely diagnostic imaging modalities and conventional ablative techniques that lack real-time biologic feedback.

This review explores how these new technologies can potentially reframe the current narrative, highlighting their individual capabilities, synergistic potential, and implications for the future of interventional pulmonology. We also examine current limitations, clinical applications, and the translational pathway required to bring this paradigm into routine practice.

## 2. The “See and Strike” Framework

The integration of Dynamic Cell Imaging (DCI) with biphasic Pulsed Electric Field (PEF) ablation introduces the possibility of a more closely integrated diagnostic–therapeutic workflow in thoracic oncology. Historically, lung cancer management has relied on sequential procedural steps that occur across multiple clinical encounters, including lesion identification, tissue acquisition, pathologic confirmation, molecular characterization, multidisciplinary discussion, cancer staging, and eventual therapeutic intervention [17]. Although advances in navigational bronchoscopy and imaging technologies have improved access to peripheral pulmonary lesions, these procedures remain primarily diagnostic and do not fundamentally alter the staged nature of the diagnostic and treatment pathway.

A persistent limitation in bronchoscopic biopsy procedures is the inability to confirm biologically viable tumor tissue at the moment of sampling. Radiographic confirmation of tool-in-lesion positioning verifies anatomical localization but does not ensure that the retrieved specimen contains diagnostically adequate malignant tissue. Current strategies, such as rapid on-site cytologic evaluation (ROSE), can improve diagnostic yield but remain dependent on cytopathology expertise and may not be universally available [18]. Consequently, nondiagnostic biopsies may occur despite technically successful procedures, leading to repeat interventions and delays in treatment initiation.

Dynamic Cell Imaging offers a potential method to address this limitation by enabling rapid visualization of cellular viability and microstructural architecture within freshly obtained biopsy specimens. Because DCI preserves tissue integrity, specimens can subsequently undergo conventional histopathology, immunohistochemistry, and molecular testing. In this context, DCI may function as an intraprocedural biologic confirmation tool that complements existing imaging modalities by verifying that the sampled tissue represents a viable tumor rather than surrounding parenchyma or necrotic debris.

PEF ablation represents a therapeutic modality that may be well-suited to act upon such confirmed biologic targets. Unlike thermal ablation technologies that rely on heat or cold to induce tissue destruction, PEF disrupts cellular homeostasis to kill cells while largely preserving extracellular matrix architecture and surrounding connective tissue structures. This non-thermal mechanism may allow treatment of lesions located near critical thoracic structures—including airways, pleura, and vasculature—where thermal ablation modalities may carry increased risk.

Within this framework, these complementary technologies could theoretically support a workflow in which viable tumor tissue is confirmed intraprocedurally, and focal therapy is delivered to the same target during a single procedural session. Following navigational access to a pulmonary lesion, tissue sampling could be evaluated using DCI to determine metabolic activity and diagnostic adequacy. Once a viable tumor is confirmed, PEF ablation could then be applied to the same lesion using bronchoscopic or percutaneous delivery systems. Although this approach remains investigational, it raises the possibility of reducing repeat diagnostic procedures and shortening the interval between diagnosis and treatment.

Future technological developments may further enhance the integration of biologic visualization and therapy [19]. For example, quantitative analysis of DCI signal patterns may eventually enable more precise identification of metabolically active tumor regions within heterogeneous lesions. Such information could potentially guide spatially targeted energy delivery during PEF ablation, conceptually analogous to biologically adaptive treatment planning strategies used in radiation oncology [20]. In addition, advances in machine learning and image analysis may facilitate automated interpretation of DCI signal patterns and decision-support systems that assist proceduralists in real-time evaluation of biopsy specimens [21].

Beyond local tumor control, there is also emerging interest in the potential immunologic effects of non-thermal ablation. Early preclinical and clinical observations suggest that PEF-induced tumor cell death may promote antigen release and immune activation while preserving surrounding stromal architecture. However, current evidence remains preliminary and derived largely from small feasibility studies and exploratory analyses [22]. Further investigation will be necessary to determine whether such immune modulation contributes meaningfully to systemic antitumor responses or improves outcomes when combined with systemic therapies.

Taken together, the convergence of DCI and PEF represents an evolving model of biologically guided intervention in lung cancer care. Rather than viewing bronchoscopy solely as a diagnostic procedure, future technological integration may enable minimally invasive platforms capable of both tumor confirmation and targeted treatment when clinically appropriate. Prospective trials and workflow optimization will be necessary to determine whether this conceptual approach can translate into improved diagnostic efficiency, procedural outcomes, and patient-centered care.

## 3. Dynamic Full-Field Optical Coherence Tomography and Dynamic Cell Imaging

Accurate real-time assessment of tissue viability during diagnostic procedures remains a persistent unmet need in thoracic oncology. Despite advances in computed tomography screening, robotic-assisted bronchoscopy, and navigational imaging platforms, confirmation of successful lesion targeting continues to rely primarily on radiographic surrogates rather than biologic validation. Conventional diagnostic pathways depend on histopathologic processing that occurs hours to days after tissue acquisition, creating a temporal disconnect between sampling and diagnostic certainty. Indeterminate or non-diagnostic biopsies contribute to repeat procedures, delayed treatment initiation, increased healthcare utilization, and patient anxiety. Full-Field Optical Coherence Tomography (FF-OCT) and Dynamic Cell Imaging (DCI) have emerged as promising technologies designed to address this gap by enabling immediate, label-free visualization of living cellular architecture and metabolic activity at the point of care [8,9,10,23,24].

FF-OCT utilizes low-coherence interferometric light to generate micron-scale images of tissue microstructure without the need for staining or exogenous contrast agents [8]. The addition of dynamic analysis in DCI extends this capability by capturing intracellular motion patterns reflecting organelle activity, cytoplasmic dynamics, and cellular viability [9]. This functional dimension distinguishes DCI from traditional optical imaging modalities that provide morphology alone. By preserving tissue integrity, DCI allows simultaneous rapid assessment of diagnostic adequacy while maintaining specimens for downstream histopathology, next-generation sequencing, and molecular biomarker testing—an increasingly critical requirement in modern precision oncology. The ability to obtain metabolic information without consuming or altering tissue represents a significant advancement over frozen section analysis or cytologic rapid on-site evaluation, both of which may be resource-intensive and limited by sampling variability.

The significance of DCI lies not only in diagnostic acceleration but also in its potential to redefine procedural decision-making. Real-time confirmation of viable tumor tissue may improve diagnostic yield, optimize sampling strategies, and reduce unnecessary biopsies, particularly in small peripheral lesions where tissue acquisition is technically challenging. Furthermore, integration of DCI into bronchoscopic workflows introduces the possibility of biologically guided intervention, forming the diagnostic foundation for emerging combined diagnostic–therapeutic paradigms. As lung cancer care increasingly shifts toward minimally invasive, precision-driven strategies, dynamic optical imaging technologies may play a central role in transforming bronchoscopy from a confirmatory diagnostic procedure into an adaptive, biologically informed interventional platform.

## 4. Equipment and Materials

Full-Field Optical Coherence Tomography (FF-OCT) and Dynamic Cell Imaging (DCI) systems are composed of an optical imaging platform designed to perform high-resolution, label-free visualization of fresh biological tissue at the point of care (see Figure 1). The Van Gogh™ system represents a clinically adapted implementation of this technology, integrating a broadband low-coherence light source, interferometric optics, high-sensitivity cameras, and computational processing software into a compact workstation suitable for procedural environments. FF-OCT operates by illuminating tissue with spatially incoherent light and detecting backscattered signals through an interferometric configuration, generating three-dimensional images with micron-scale resolution comparable to histologic sections. Unlike confocal microscopy or fluorescence-based imaging, FF-OCT does not require staining, fixation, or exogenous contrast agents, allowing freshly obtained biopsy specimens to be analyzed immediately after acquisition. Tissue samples obtained via bronchoscopic forceps biopsy, needle aspiration, cryobiopsy, or surgical excision are placed directly onto a sterile sample holder or imaging cartridge, typically under gentle coverslip compression to stabilize the specimen and optimize optical signal acquisition.

DCI builds upon the structural imaging capability of FF-OCT through computational analysis of temporal fluctuations in light scattering caused by intracellular organelle motion [8]. Proprietary software algorithms analyze dynamic signal variations across sequential image frames, producing functional maps that reflect metabolic activity and cellular viability. The imaging workflow is designed for rapid integration into bronchoscopy or interventional suites, with acquisition times typically ranging from seconds to minutes, thereby enabling near real-time feedback regarding tissue adequacy. Importantly, imaging is non-destructive, permitting the same specimen to proceed to formal histopathology, immunohistochemistry, or next-generation sequencing without compromise. Supporting materials include sterile disposable sample interfaces, vibration-isolated imaging stages to minimize motion artifact, and integrated computational hardware capable of high-throughput image reconstruction and analysis. Collectively, these components allow FF-OCT and DCI systems to provide a structural and functional three-dimensional image to function as an intra-procedural digital microscopy platform, bridging conventional pathology and interventional imaging while maintaining compatibility with existing diagnostic workflows.

## 5. Biologic Validation and Diagnostic Performance of DCI

Dynamic Cell Imaging (DCI) is a label-free optical imaging modality that enables real-time visualization of cellular metabolic activity and microstructural morphology without tissue destruction. Unlike conventional histopathology, which requires fixation, staining, and processing time, DCI captures intrinsic intracellular motion signatures generated by organelle dynamics, allowing differentiation of viable from non-viable tissue while preserving specimens for downstream molecular testing. Foundational biologic validation by Park et al. demonstrated that dynamic FF-OCT (DCI) could distinguish live from apoptotic HeLa cells based on distinct frequency-domain motion patterns, establishing DCI as a functional imaging platform rather than purely structural microscopy [8]. This study confirmed that metabolic activity can be quantitatively inferred from intrinsic optical signals, supporting its translational application in oncologic diagnostics.

Subsequent clinical investigations have expanded the evidence base for FF-OCT and DCI across multiple malignancies [8,9,10,25,26]. As summarized in Table 1, studies in breast, dermatologic, gastrointestinal, prostate, gynecologic, and thoracic oncology consistently demonstrate high concordance with final histopathology, with reported diagnostic sensitivities ranging from 66.7 to 94%, specificities of 64–100%, and overall accuracy approaching 96% [9]. Although many of these investigations are single-center and non-randomized, the reproducibility of diagnostic performance across organ systems strengthens the biologic plausibility and clinical applicability of this imaging platform.

In thoracic oncology specifically, Matus et al. evaluated proceduralist-directed rapid on-site pathologic evaluation (ROPE) using DCI during robotic-assisted bronchoscopy for peripheral pulmonary lesions [10]. In this pilot cohort of 50 patients and 146 biopsies, DCI achieved 88% concordance with final pathology and identified lesional tissue in cases initially interpreted as inconclusive by conventional assessment, while maintaining tissue integrity for molecular profiling. Collectively, the data presented in Table 1 suggest that DCI may bridge the gap between radiographic tool confirmation and biologic tumor verification by introducing real-time functional validation into bronchoscopic biopsy workflows.

## 6. Pulsed Electric Field (PEF) Ablation

Aliya^®^ Pulsed Electric Field (PEF) ablation is a non-thermal therapeutic modality that induces tumor cell death through the application of high-voltage electrical pulses rather than temperature-mediated tissue destruction [11,12,13,14,15]. Unlike conventional ablative approaches such as radiofrequency, microwave, or cryoablation, PEF disrupts cellular homeostasis by transiently altering transmembrane potentials.

Because this mechanism primarily influences cellular membranes rather than extracellular structural proteins, connective tissue scaffolding, collagen matrices, vasculature, and airway architecture remain largely preserved [11,15]. This characteristic distinguishes PEF from thermal ablation technologies and enables treatment of lesions located near critical thoracic structures where thermal approaches may carry substantial risk.

In thoracic oncology, these properties make PEF particularly attractive for tumors adjacent to bronchi, pleura, mediastinal structures, or major vessels. Modern PEF platforms utilize catheter-based delivery systems that can be introduced through either percutaneous or bronchoscopic approaches. Once positioned within target tissue, short-duration biphasic high-voltage pulses are delivered, producing predictable ablation zones defined by electrical field distribution rather than thermal conduction. This approach allows localized cellular depletion while maintaining structural integrity of surrounding tissue.

Beyond direct cytoreduction, emerging data suggest that PEF may also influence the tumor microenvironment by promoting immunogenic cell death and antigen release, potentially augmenting systemic immune responses when combined with contemporary oncologic therapies.

## 7. Equipment and Materials

PEF ablation systems consist of three primary components: an electrical pulse generator, a catheter-based delivery system, and imaging guidance platforms for procedural navigation. The Aliya PEF system (Galvanize Therapeutics, Redwood City, CA) employs a monopolar biphasic waveform generator capable of delivering high-voltage electrical pulses in controlled sequences designed to induce regulated cell death processes.

Energy delivery occurs through a flexible catheter incorporating a deployable needle electrode capable of penetrating target tissue under bronchoscopic, navigational bronchoscopy, or percutaneous guidance (Figure 2, Figure 3 and Figure 4). The catheter connects to an external pulse generator that precisely regulates pulse amplitude, duration, and frequency to achieve reproducible ablation zones while minimizing electrical or thermal injury to surrounding structures.

Procedural guidance typically involves navigational bronchoscopy, fluoroscopy, or cone-beam CT imaging to confirm electrode positioning prior to energy delivery. Because PEF ablation does not rely on thermal gradients, temperature monitoring probes and cooling systems required by microwave or radiofrequency platforms are unnecessary. Additional system components include sterile disposable electrode assemblies, grounding interfaces, and integrated monitoring software that verifies appropriate energy delivery and safety parameters during treatment.

## 8. Biological Validation and Translational Evidence

Aliya PEF ablation is an emerging focal modality that kills cells without reliance on temperature changes. It has been used for lung lesions in patients with metastatic cancer [12,13]. In addition to percutaneous approaches, the system also includes a flexible endoluminal needle that can deliver PEF energy to target tissues using conventional bronchoscopy or navigational bronchoscopy (see Figure 2 and Figure 3). The ablation procedure is performed using a monopolar needle that delivers a preset PEF dose. Ablation delivers a series of pulses that are short duration, biphasic high voltage to alter transmembrane potentials, resulting in an accumulated disruption of homeostasis that results in the induction of regulated cell death mechanisms (see Figure 3, Figure 4 and Figure 5). The biphasic waveform also minimizes the potential for arrhythmogenicity, comparable to the findings shown by GR Meininger and colleagues [14]. This does not rely on thermal gradients. In contrast to the thermal ablation modalities, this approach preserves connective tissue, pleura and at-risk sensitive structures like vasculature and airways as shown in porcine models and published clinical experience [15].

Kaviani et al. evaluated liver, kidney, and skeletal muscle in porcine models using biphasic monopolar PEF ablation and demonstrated predictable focal ablation zones with preservation of extracellular matrix architecture [16]. Serial imaging showed radiographic evolution without collateral structural damage. No electrocardiographic disturbances were observed during delivery. Preservation of the stromal matrix is a distinguishing feature of PEF compared to thermal modalities.

## 9. Safety near Critical Thoracic Structures

The major concerns about ablative modalities are safety for critical structures. Hunter et al. demonstrated that when PEF was delivered within millimeters of the critical structures in the chest (pleura, airways, and vasculature), histology showed cellular depletion zones with intact connective tissue scaffolding. The first in-human validation was shown in the INCITE-ES treat-and-resect trial of early-stage NSCLC [27]. In this trial, 36 patients underwent PEF ablation at the time of diagnostic biopsy, followed by their regularly scheduled surgical resection approximately 3 weeks later. There were no reported device-related serious adverse events and no delays or accommodations needed for the surgical approach. Like the preceding studies, histopathology confirmed cellular depletion zones, with good consistency of ablation zone regardless of proximity to pleura, blood vessels, or major airways. Further, histological confirmation demonstrated intact sensitive structures, including pleura, airways, and major vasculature. Tertiary lymphoid structures were observed to be preserved in the treated specimens. This study supported feasibility ahead of surgical interventions, as clinical observations showed that ablation adjacent to mediastinal structures did not result in significant adverse sequelae. These findings have important implications in operable lung cancer, where proximity to adjacent vital structures can pose challenges. However, the intentionally incomplete ablation design precludes definitive conclusions about efficacy.

PEF can also benefit cases of systemic therapies, potentially through an abscopal-like effect. The AFFINITY feasibility trial evaluated PEF at the time of diagnostic biopsy prior to initiation of systemic therapy [28]. 30-day safety data showed acceptable tolerance and no interference with planned systemic therapies. 6-month data showed local control in the ablation-only subgroup and detection of circulating immune cell populations as well as tumor-associated antigen-specific IgG responses. This was not a randomized study but raises important questions about the possibility of immunogenic modulation with PEF. Finally, the addition of this ablation to standard of care in Stage IV metastatic NSCLC patients that had progressed after first-line therapy resulted in increases in 1-year progression-free survival (from 12% to 63%) and overall survival (from 33% to 74%) compared to a cohort-matched population that did not receive ablation.

## 10. Limitations

Despite the promising conceptual and translational rationale for these technologies, several important limitations warrant careful consideration. First, the current evidence base supporting both Dynamic Cell Imaging (DCI) and Pulsed Electric Field (PEF) ablation efficacy remains largely early phase, consisting primarily of single-center studies, feasibility trials, and small prospective cohorts. Sample sizes are modest, follow-up durations are limited, and most investigations are non-randomized without direct comparison to standard-of-care modalities such as rapid on-site cytologic evaluation (ROSE), thermal ablation, stereotactic body radiation therapy, or surgical resection. As a result, superiority, non-inferiority, or long-term oncologic equivalence cannot yet be established. Reported diagnostic concordance and short-term safety outcomes are encouraging, but durability of local control, progression-free survival, and overall survival data are not yet mature.

Second, workflow integration remains an unresolved practical challenge. Incorporating DCI into bronchoscopic procedures introduces additional steps that may affect procedural duration, staffing requirements, and operator learning curves. Inter-reader variability in interpretation of metabolic imaging patterns has not been fully characterized, and standardized training or certification frameworks do not yet exist. Similarly, while PEF avoids thermal injury, optimal dosing parameters, energy delivery algorithms, and post-ablation imaging criteria remain incompletely defined. Radiographic evolution following non-thermal ablation may differ from traditional modalities, potentially complicating response assessment and surveillance strategies.

Third, regulatory and reimbursement landscapes are still evolving. PEF currently carries device-level clearances rather than disease-specific indications in many regions, and widespread adoption will depend on demonstration of cost-effectiveness and alignment with payer models. Capital investment, disposable device costs, and maintenance infrastructure may limit accessibility in resource-constrained systems. Additionally, several early studies involve industry collaboration or sponsorship, which, while common in device development, may introduce potential bias and underscore the need for independent multicenter validation.

Finally, the proposed immunologic synergy between PEF and systemic therapy remains hypothesis-generating rather than definitive. Evidence of immune activation is preliminary and derived from exploratory analyses without randomized controls. The biologic heterogeneity of lung cancer, including variable tumor microenvironments and molecular subtypes, may influence response to both DCI-guided targeting and PEF-induced cell death. Accordingly, careful patient selection criteria and biomarker-driven trial design will be essential. Until robust, comparative, long-term data are available, this paradigm should be viewed as an evolving investigational strategy rather than an established standard of care.

## 11. Conclusions and Future Directions

The integration of Dynamic Cell Imaging (DCI) and biphasic Pulsed Electric Field (PEF) ablation represents an emerging approach toward real-time, biologically guided bronchoscopic intervention in thoracic oncology. Together, these technologies may enable intraprocedural confirmation of viable tumor tissue followed by immediate focal, non-thermal therapy within a single procedural workflow. This paradigm has the potential to reduce repeat procedures, shorten time to treatment, and expand minimally invasive treatment options for patients with early-stage disease, oligometastatic lesions, or tumors located near critical thoracic structures.

DCI offers rapid, label-free assessment of tissue viability while preserving specimens for downstream histopathology and molecular profiling, whereas PEF provides structurally sparing tumor ablation without reliance on thermal injury. Early translational and clinical data support the feasibility and safety of both technologies, particularly in anatomically sensitive thoracic environments.

Despite these promising findings, current evidence remains limited to early-phase studies with modest sample sizes and short follow-up durations. Prospective multicenter trials will be necessary to determine diagnostic accuracy, durability of local tumor control, long-term oncologic outcomes, workflow integration, and cost-effectiveness relative to established standards of care.

As with other emerging bronchoscopic technologies such as robotic bronchoscopy and augmented imaging platforms, adoption of DCI and PEF will likely occur progressively as clinical evidence, technological refinement, and procedural familiarity continue to evolve [26]. As lung cancer care increasingly shifts toward precision-based, minimally invasive intervention, the integration of biologic imaging and non-thermal ablation may help redefine bronchoscopy from a predominantly diagnostic procedure to a more integrated diagnostic–therapeutic platform.

## Figures and Tables

**Figure 1:**
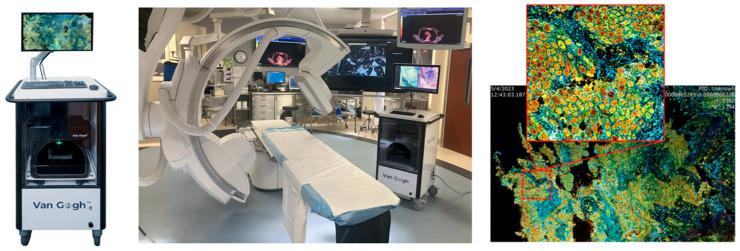
Dynamic full-field optical coherence tomography (FF-OCT) and Dynamic Cell Imaging (DCI) systems are composed of an optical imaging platform designed to perform high-resolution, label-free visualization of fresh biological tissue at the point of care (**Left**). Workflow integration of Van Gogh with current bronchoscopic imaging and navigation systems (**Middle**). Dynamic Cell Image of a Lung Nodule Cryobiopsy. Representative DCI image of a lesional lung tissue obtained through cryobiopsy. The DCI system uses phase contrast microscopy with Michelson interferometry to visualize metabolic activity (**Right**).

**Figure 2:**
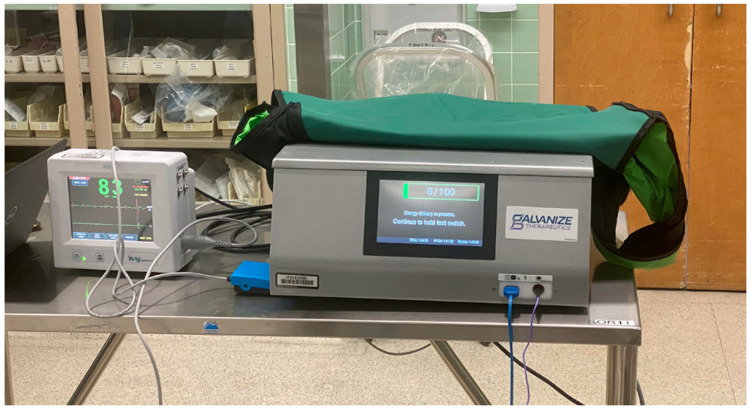
PEF (Galvanize) set up with electrocardiograph.

**Figure 3:**
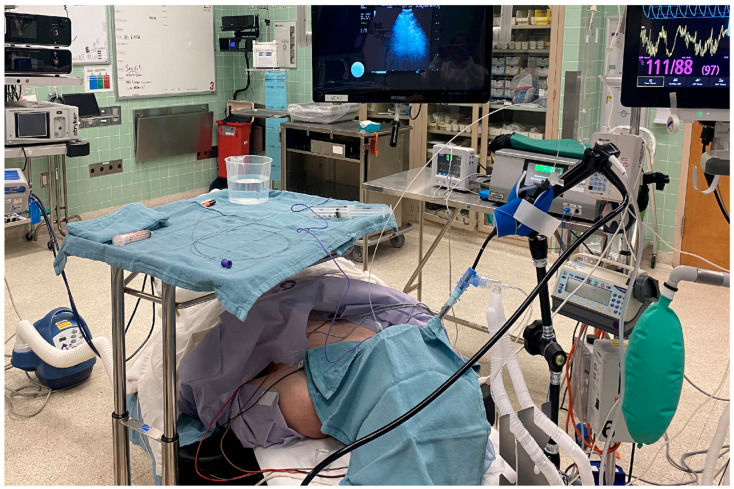
Procedural Photo Utilizing Aliya Electrode (Galvanize) for PEF delivery via convex endobronchial ultrasound (EBUS). Photo illustrates the patient under general anesthesia with an EBUS scope held in position with a scope holder to deliver PEF.

**Figure 4:**
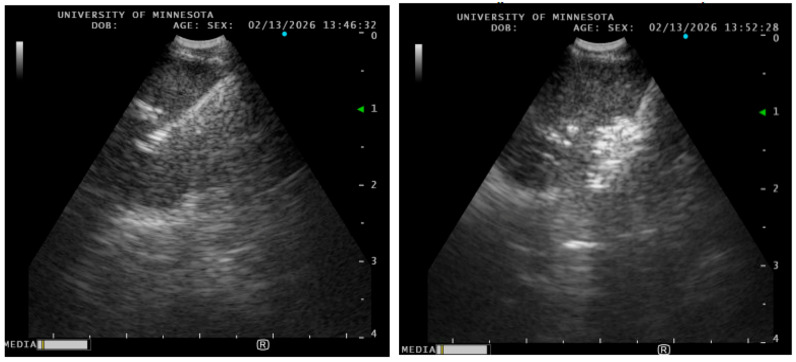
Convex EBUS was used to access the Station 7 lymph node (**left**) and shown here are the pre- and post- activation images from 1 vector with post-ablation changes seen (**right**).

**Figure 5:**
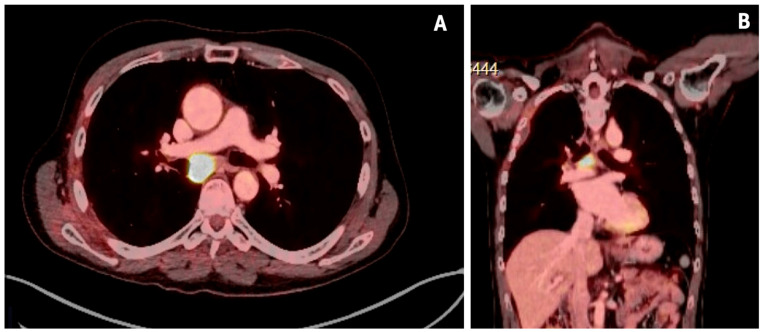
PET-CT of the Metastatic Lymph Node. The PET-CT confirmed the subcarinal lymph node involvement and the decision was to target this for ablation. Both axial (**A**) and Coronal (**B**) sections were included in the measurements to calculate treatment dose.

**Table 1:** Landmark studies in the validity of dynamic FF-OCT.

Study	Design	Comparison Groups	Diagnostic Performance	Molecular Impact	Operational Impact	Clinical Implications
Park et al. [8]	Experimental study with dynamic full-field OCT and machine learning	Live versus dead cells (n = 3404 cells)	Balanced accuracy 94%; sensitivity 98% (for live cells)	Confirms metabolic viability detection	Non-destructional (preserves tissue for downstream histopathology)	Biologic in-vivo basis for real-time viability. Label-free differentiation of live and dead cells
Pavone et al. [9]	Systematic review of 31 studies	Full-field OCT guided DCI performance across organ systems	Sensitivity 94%; specificity close to 100%	Multiple organ systems were validated	Rapid adjunct to histopathology	Data shows DCI performance is organ-agnostic
Matus et al. [10]	Prospective single-center study	DCI concordance with final path in RAB (n = 50 patients)	88% concordance and 100% sensitivity	Flagged lesional tissue in 4 cases that were initially conclusive and later confirmed malignant	Real-time lesion confirmation	Reduced false negatives
Deitz et al. [23]	Retrospective matched cohort	DCl (n = 94) versus ROSE (n = 364) during RAB	Accuracy of 98% (vs. 64% with ROSE). 61% reduction of indeterminate cases (4.3% vs. 11%)	Statistically non-inferior to ROSE. 13% of the cases initially reported negative were confirmed malignant on final pathology	Procedure time shortened by ~11 min	Improved intraprocedural diagnostic confidence
Manley et al. [24]	Multi-center prospective pilot for DCI sensitivity and work.flow integration in RAB	Comparison to ROSE (n = 54)	Sensitivity 90% (ROSE 44%) Specificity 77% (ROSE 100%)	Higher sensitivity	Preparation time was <2 minutes	Deployment of Van Gogh™ is feasible with higher sensitivity but lower specificity
Nemeh et al. [25]	Prospective comparison of tissue adequacy for NGS following DCI	Tissue adequacy and concordance forNCS following DCI in final pathology (n = 51 patients; 24 malignant on final pathology)	n/a	100% sufficiency for NGS testing among malignant samples (all 23 samples evaluated were deemed sufficient for molecular profiling)	Non-destructional (preserves tissue for downstream histopathology)	Tissue preserved for comprehensive genomic profiling

## Data Availability

No new data were created or analyzed in this study. Data sharing is not applicable to this article.

## Abbreviations

Computed Tomography (CT); Dynamic Cell Imaging (DCI); Endobronchial Ultrasound (EBUS); Non-Small Cell Lung Carcinoma (NSCLC); Optical Coherence Tomography (OCT); Pulsed Electric Field (PEF); Peripheral Lung Lesions (PPL); Robotic Assisted Bronchoscopy (RAB); Rapid On-Site Pathologic Evaluation (ROPE); Rapid On-Site Evaluation (ROSE); Stereotactic Body Radiation Therapy (SBRT).

## References

[1] Weickhardt A.J., Scheier B., Burke J.M., Gan G., Lu X., Bunn P.A., Aisner D.L., Gaspar L.E., Kavanagh B.D., Doebele R.C. (2012). Local ablative therapy of oligoprogressive disease prolongs disease control by tyrosine kinase inhibitors in oncogene-addicted non–small-cell lung cancer. J. Thorac. Oncol..

[2] American Cancer Society (2021). Cancer Facts & Figures 2021. https://www.cancer.org/content/dam/cancer-org/research/cancer-facts-and-statistics/annual-cancer-facts-and-figures/2021/cancer-facts-and-figures-2021.pdf.

[3] Stella G.M., Kolling S., Benvenuti S., Bortolotto C. (2019). Lung-seeking metastases. Cancers.

[4] Qi H., Fan W. (2018). Value of ablation therapy in the treatment of lung metastases. Thorac. Cancer.

[5] Thompson M., Rosenzweig K.E. (2019). The evolving toxicity profile of stereotactic body radiation therapy for lung cancer. Transl. Lung Cancer Res..

[6] Milano M.T., Chen Y., Katz A.W., Philip A., Schell M.C., Okunieff P. (2009). Central thoracic lesions treated with hypofractionated stereotactic body radiotherapy. Radiother. Oncol..

[7] Hiraki T., Gobara H., Fujiwara H., Ishii H., Tomita K., Uka M., Makimoto S., Kanazawa S. (2013). Lung cancer ablation: Complications. Semin. Interv. Radiol..

[8] Park S., Nguyen T., Benoit E., Sackett D.L., Garmendia-Cedillos M., Pursley R., Boccara C., Gandjbakhche A. (2021). Quantitative evaluation of the dynamic activity of HeLa cells in different viability states using dynamic full-field optical coherence microscopy. Biomed. Opt. Express.

[9] Pavone M., Innocenzi C., Carles E., Bizzarri N., Moro F., Ferrari F.A., Nicolae A., Testa A.C., Fanfani F., Fagotti A. (2025). Cutting-edge microscopic intraoperative tissue assessment for guidance in oncologic surgery: A systematic review of the role of optical coherence tomography. Ann. Surg. Oncol..

[10] Matus I., Akhtar S., Raja H., Holmes P. (2026). Proceduralist-directed rapid on-site pathologic evaluation using dynamic cell imaging: A pilot study of peripheral pulmonary lesion sampling through robotic bronchoscopy. J. Bronchol. Interv. Pulmonol..

[11] Batista Napotnik T., Polajzer T., Miklavčič D. (2021). Cell death due to electroporation: A review. Bioelectrochemistry.

[12] Moore W.H., Silk M., Bhattacharji P., Pua B.B., Mammarappallil J., Sterman D.H., Chachoua A. (2025). Early experience with pulsed electric field ablation in the setting of recalcitrant stage IV lung cancer. Lung Cancer.

[13] Moreno-Gonzalez A., Nafie E.H.O., Pastori C., Mammarappallil J., Seshaiah P., Plentl M.B., Hatton B.A., Neal R.E., Pritchett M.A., Reisenauer J.S. (2025). Six-month local control rates and immune responses after pulsed electric field ablation in metastatic cancer. Cancers.

[14] Meininger G.R., Neal R.E., Hunter D.W., Krimsky W.S. (2023). Absence of arrhythmogenicity with biphasic pulsed electric fields delivered to porcine airways. Ann. Biomed. Eng..

[15] Hunter D.W., Krimsky W., Meyerhoff R.R., Mammarappallil J.G., Kaviani R., Stoffregen W.C., Reisenauer J. (2025). Pulsed electric field ablation safety and characterization near sensitive structures in lung: A preclinical and clinical case series study. J. Thorac. Dis..

[16] Kaviani R., Neal R.E., Hunter D.W., Krimsky W.S. (2024). Tissue radiologic and pathologic response to biphasic pulsed electric field technology in a porcine model. J. Vasc. Interv. Radiol..

[17] Rivera M.P., Mehta A.C., Wahidi M.M. (2013). Establishing the diagnosis of lung cancer: Diagnosis and management of lung cancer, 3rd ed: American College of Chest Physicians evidence-based clinical practice guidelines. Chest.

[18] Munoz M.L., Lechtzin N., Li Q.K., Wang K., Yarmus L.B., Lee H.J., Feller-Kopman D.J. (2017). Bronchoscopy with endobronchial ultrasound guided transbronchial needle aspiration vs. transthoracic needle aspiration in lung cancer diagnosis and staging. J. Thorac. Dis..

[19] Rubinsky B. (2007). Irreversible electroporation in medicine. Technol. Cancer Res. Treat..

[20] Green O.L., Henke L.E., Hugo G.D. (2019). Practical Clinical Workflows for Online and Offline Adaptive Radiation Therapy. Semin. Radiat. Oncol..

[21] Esteva A., Robicquet A., Ramsundar B., Kuleshov V., DePristo M., Chou K., Cui C., Corrado G., Thrun S., Dean J. (2019). A guide to deep learning in healthcare. Nat. Med..

[22] Moore W.H., Silk M., Bhattacharji P., Pua B.B., Mammarappallil J., Meyerhoff R.R., Kessler J., Tasse J., Gulizia D. (2025). Safety and feasibility of percutaneous pulsed electrical field ablation in multiple organs: A multi-center retrospective study. Eur. J. Radiol..

[23] Deitz R., Ryan J., Levy R., Awais O., Kovacs S. Dynamic cell imaging as an alternative to rapid on-site evaluation in robotic bronchoscopy: A comparative study. Presented at the American Thoracic Society International Conference.

[24] Manley C., Parsons M., Cerda S., Shevtsov A., Gong Y., Ehya H., Laxmanan B., Billatos E. Multicenter prospective study of a novel biopsy assessment system for the intraprocedural evaluation of lung biopsies by robotic-assisted bronchoscopy. Presented at the American Thoracic Society International Conference.

[25] Nemeh C.N., Wagh A.A., Mueller J., Wagh A.A. Use of the Van Gogh™ microscopy system to assess intra-procedural tissue adequacy: Initial experience. Presented at the American Thoracic Society International Conference.

[26] Cho R.J., Senitko M. (2023). The Road to 90. J. Bronchol. Interv. Pulmono..

[27] Jimenez M., Flandes J., van der Heijden E.H.F.M., Ng C.S.H., Iding J.S., Garcia-Hierro J.F., Recalde-Zamacona B., Verhoeven R.L.J., Lau R.W.H., Moreno-Gonzalez A. (2025). Safety and feasibility of pulsed electric field ablation for early-stage non-small cell lung cancer prior to surgical resection. J. Surg. Oncol..

[28] Pritchett M.A., Reisenauer J.S., Fernandez-Bussy S., DiBardino D., Hunt S.J., Ma K.C., Pua B.B., Shostak E., Hatton B.A., Moreno-Gonzalez A. (2025). The safety of pulsed electric field ablation before standard-of-care treatment for patients with metastatic cancer. J. Bronchol. Interv. Pulmonol..

